# Online 3-Dimensional Path Planning with Kinematic Constraints in Unknown Environments Using Hybrid A* with Tree Pruning

**DOI:** 10.3390/s21041152

**Published:** 2021-02-06

**Authors:** Jonatan Scharff Willners, Daniel Gonzalez-Adell, Juan David Hernández, Èric Pairet, Yvan Petillot

**Affiliations:** 1 Institute of Sensors, Signals and Systems, Heriot-Watt University, Edinburgh EH14 4AS, UK; dg36@hw.ac.uk (D.G.-A.); y.r.petillot@hw.ac.uk (Y.P.); 2 Centre for Artificial Intelligence, Robotics and Human-Machine Systems (IROHMS), Cardiff University, Cardiff CF24 3AA, UK; hernandezvegaj@cardiff.ac.uk; 3 Edinburgh Centre for Robotics, University of Edinburgh and Heriot-Watt University, Edinburgh EH14 4AS, UK; eric.pairet@ed.ac.uk

**Keywords:** hybrid A*, autonomous underwater vehicle, path planning, unknown environments, graph-search, online replanning, tree pruning

## Abstract

In this paper we present an extension to the hybrid A* (HA*) path planner. This extension allows autonomous underwater vehicle (AUVs) to plan paths in 3-dimensional (3D) environments. The proposed approach enables the robot to operate in a safe manner by accounting for the vehicle’s motion constraints, thus avoiding collisions and ensuring that the calculated paths are feasible. Secondly, we propose an improvement for operations in unexplored or partially known environments by endowing the planner with a tree pruning procedure, which maintains a valid and feasible search-tree during operation. When the robot senses new obstacles in the environment that invalidate its current path, the planner prunes the tree of branches which collides with the environment. The path planning algorithm is then initialised with the pruned tree, enabling it to find a solution in a lower time than replanning from scratch. We present results obtained through simulation which show that HA* performs better in known underwater environments than compared algorithms in regards to planning time, path length and success rate. For unknown environments, we show that the tree pruning procedure reduces the total planning time needed in a variety of environments compared to running the full planning algorithm during replanning.

## 1. Introduction

Marine robots have revolutionised our understanding of the marine environment and our ability to access and exploit it as they can venture far beyond where humans can in this extreme environment. As of today, remotely operated vehicle (ROVs) are still the norm in many underwater applications but they are limited as they require a support ship, an operator and a tether to provide power and control to the system. Autonomous marine robots are now a mature technology for survey and their autonomy (and capabilities) are rapidly increasing, enabling them to operate for longer periods, with both less and more efficient human supervision [1], and even the possibility to be permanently deployed using underwater docking and charging stations [2].

The ability to guarantee precise and robust trajectories is a critical capability for most robots, however, this is particularly challenging for underwater vehicles because, in general, they are non-holonomic and subject to unpredictable external forces such as currents and waves. Moreover, underwater robots often operate in unknown environments and they have to adapt their path to the terrain (including obstacles) online. Collisions in the maritime domain can have serious consequences as damage to the vehicle might lead to leakage, internal damage of the hardware and, in the worst-case scenario, loss of the asset.

### 1.1. Related Work

Motion planning of traditional robots is a widely researched area [3]. In the maritime domain, the motion planning problem is less extensively researched and most planners consider only geometric constraints. For a mobile robot to operate autonomously it needs to be able to find a solution to a start-to-goal query. This solution helps the robot to navigate from a start configuration to a goal configuration or region. A valid solution needs to be collision-free in the configuration space (C-Space) (a space that contains all possible configuration of the robot) [4]. The C-Space includes position in *n* dimensions, noted as $R^{n}$. The orientation of a C-Space is noted as SO(n). For both position and orientation this is noted as SE(n)=$R^{n}$×SO(n). For an underwater robot moving in three dimensions, the C-Space would have 6 dimensions—SE(3)= [x, y, z, roll (φ), pitch (θ), yaw (ψ)]. Table 1 presents an overview of some of the previous work with emphasis on the capabilities relating to C-Space, kinematic constraints, online planning and if the approach was used with autonomous underwater vehicles (AUVs).

Widely used approaches for path planning are search-based or grid-based methods such as Dijkstra’s Algorithm [5] and A* [6]. Grid-based methods create a discrete grid of the C-Space and apply graph search strategies to find a path that leads to a given goal configuration. The grid-based methods are fast and often find the optimal path based on the resolution of the grid. However, they do not necessarily scale well with the size and dimension of the C-Space. These methods are resolution-complete—if a solution exists in the discrete representation of the C-Space, the algorithm will find it. However, the discretisation balances computing requirements with accuracy and can lead to non-optimal solutions or failures if the grid is too coarse (e.g., for dealing with narrow passages). A grid-based approach presented by Tanakitkorn et al. [7] use a genetic algorithm (GA) that finds shorter paths than A* but with a much longer planning time.

A family of algorithms which does not rely on discretising the C-Space are sampling-based methods, or stochastic methods, such as probabilistic roadmap (PRM) [8] and rapidly-exploring random tree (RRT) [9]. These methods rely on randomly sampling configurations from the C-Space and connecting them to each other. By not relying on a discrete grid to perform the search, sampling-based approaches are not limited by the resolution of a grid in the same way as search-based methods are. This, however, comes with the drawback of optimality and completeness. Sampling-based methods are probabilistic complete meaning that, if a solution exists, the probability of finding it rises with search time with a limit of 1. However, they cannot guarantee that they will find a solution over a limited number of iterations. Later, extensions such as the asymptotically optimal RRT (RRT*) [10] were developed to overcome the optimality issues by introducing a re-wiring step to improve the path. The solution of this method will converge towards the optimal as the number of iterations goes towards infinity. A drawback of sampling-based algorithms is the lack of guarantees to find a solution and optimality.

**Figure 1:**
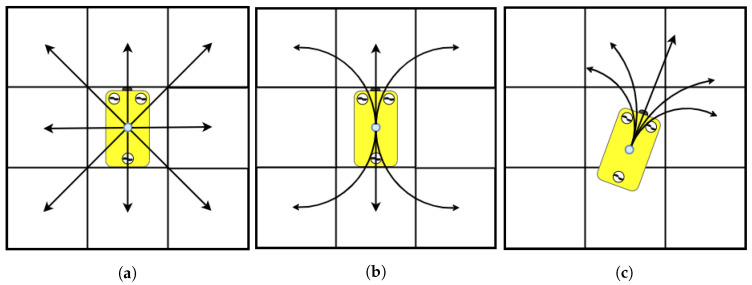
(**a**) A* and Dijkstra’s algorithm use discrete states in the centre of a cell. (**b**) State-lattice A* can connect the centre of cells using curvatures and straight lines [12]. (**c**) Hybrid A* (HA*) uses continuous values for states within cells instead of discrete location of states. This allows for smooth paths which can utilise a larger configuration space (C-Space) than (**a**,**b**).

For non-holonomic robots, finding a collision-free path is not sufficient. The path also needs to take the motion constraints of the vehicle into consideration. The original form of grid-based methods such as Dijkstra’s and A* do not take this into account. An approach by Yan et al. [11] uses A* with a circular search to ensure that the path is feasible for an AUV. Pivtoraiko et al. [12] extended A* to connect states based on Reeds–Shepp curves with the condition that the configurations are in the centre of the cells and that their orientations are contained in a discrete set, see Figure 1b. Another approach to solving the path planning problem under motion constraints with a search-based method is the hybrid A* (HA*) [13]. HA* allows states to have continuous values within the cells as can be seen in Figure 1c. It has mainly been used for terrestrial vehicles operating in SE(2).

There are multiple sampling-based algorithms which consider the motion constraints of the robot while planning a path. RRT was initially developed for kinematic planning [9]. expansive space trees (EST) [14] perform random sampling from a random state to explore the search space with kinematic constraints. EST was later extended to use a guided method to select what state to expand, to solve the query in fewer iterations [15]. Hernández et al. added Dubins curves [16] to enable RRT* to be used with kinematic constraints [17]. However, a major drawback of Dubins curves is that it only considers one radius for the curvature of the trajectory. stable sparse-RRT (SST) has been used for an AUV operating in SE(2) by Pairet et al. [18]. SST has also been used to bias the sampling to follow a lead calculated by RRT* [19,20]. Jian et al. use RRT* to find a global path in 2D which is then followed by dynamic window approach (DWA) while including the motion constraints of the AUV in SE(2) [21].

The majority of the underwater domain remain unmapped, hence robots need to be capable of responding to new information about the environment. As such, if the environment contains structures or natural obstacles, the robot should preferably be able to both sense them and adjust the plan to avoid collisions. For terrestrial robots, dynamic A* (D*) [30], Field-D* [22] and anytime dynamic A* (ADA*) [24] have been proposed and successfully used thanks to their ability to handle changes in the environment without re-calculating a new solution from an empty search tree. However, these approaches are not suitable for non-holonomic vehicles as they do not take kinematic constraints into account. Instead, the majority of the path planning approaches that consider the kinematics of the vehicle discards the tree to replan a new path when the current path is no longer valid. As an alternative approach to discarding the tree, Bekris and Kavraki [31] proposed to keep the paths that lead to the goal. This was used with a sampling-based planner guided by a heuristic which incorporates the kinematics of the robot and hence can incrementally improve the solution and potentially use other solutions in replanning. Another approach to improve replanning time is through pruning the tree of infeasible paths when new obstacles are sensed which have been used with Dynamic-RRT by Ferguson et al. [32]. Tree pruning was also used with kinematic based paths with RRT* to reuse the last best known solution for an AUV [17,33].

In this paper, we present an extension to HA* to plan feasible and collision-free paths for underwater robots operating in SE(3). Insight in the proposed extension is given in Section 2. Furthermore, in Section 3, we endow the planner with a tree pruning procedure which updates and maintains a valid search tree during operation in unexplored environments. The tree pruning step enables the reuse of previously explored paths, leading to improved on-board online replanning capabilities. HA* is resolution complete, just as A* and state-lattice A*, but HA* is able to utilise more of the discrete space due to using continuous values. HA* has an advantage over probabilistic complete approaches, as HA* is able to report if no solution exists. If the query cannot be solved, this can be reported and handled at a higher level in the autonomy. Our approach is motivated by the fact that many AUVs have non-holonomic motion constraints. Work such as RRT* with Dubins in SE(3), which includes replanning capabilities [17], is limited by constant turning radius based on the surge speed of the robot and is limited to AUVs with hovering capabilities to operate in 3D. However, many types of AUVs are required to adjust their pitch angle to change depth, for these vehicles this dimension should be included during planning. We show the efficiency of the algorithm in both known and unexplored environments in Section 4. Finally, we conclude the paper in Section 5.

### 1.2. Statement of Contributions

The main contributions of this manuscript can be summarised as follows:

- Extending HA* for robots operating in SE(3). The approach is focused on AUVs and includes domain-related constraints.
- Improved HA* operation in unexplored environments by applying a tree pruning procedure which maintains a valid search tree that can be reused when replanning is needed.
- Our proposed approach shows improved results in known environments regarding planning time, success rate and path length (quality of solution) compared to state-lattice A*, RRT and RRT* with Dubins curves.
- For unexplored environments, we show a consistent reduction in planning time by using the tree pruning procedure compared to discarding the tree and planning from scratch.

## 2. Hybrid-A* for the Underwater Domain

HA* was initially used for autonomous cars by Dolgov et al. [13,26], who experimentally showed that HA* is capable of planning in low enough time for online operation in SE(2). In this section, we will describe our adaptation of the algorithm to enable operation in SE(3) and the underwater domain. However, underwater robots, compared to terrestrial, are often equipped with less computational power as the physical space for computers and payloads is severely limited. This often means that a single, or in some cases a couple of computers, need to handle all computations from low-level control to mapping, planning and data acquisition (e.g., SPARUS II [34,35], Girona500 [36] and Iver3 (https://auvac.org/files/uploads/platform_pdf/iver3_auv_brochure.pdf (accessed on 6 February 2021)) are equipped with one computer in the basic form while ASTERx, which is a larger AUV, is endowed with two computers (https://www.eurofleets.eu/vessel/auv-asterx-or-idefx/ (accessed on 6 February 2021)). Hence, relieving the computer of additional computational expense during planning is desirable. To improve the usage of HA*, in Section 3 we present our proposed extension to reduce planning time in unexplored environments by using tree pruning to maintain a feasible and collision-free search-tree that can be used for replanning.

### 2.1. Hybrid-A*

HA* [13] is an extension to A* [6] which, instead of discrete values, uses continuous values to represent the configuration of the robot. Both algorithms are part of grid-based search methods, which discretise the search space into a grid. The grid is constructed of cells. A* store states in the centre of the cells of the grid. In HA*, states are instead stored as continuous values within the cell [13]. This representation allows for continuous movement based on the kinematic of the vehicle within the search space, enabling HA* to find paths that are feasible (doable) for non-holonomic vehicles.

The HA* algorithm (see Algorithm 1) is based around using a priority queue, which is commonly referred to as the *open list*. The *open list* contains the states which have not yet been expanded and can lead to a possible solution. In each iteration, the algorithm removes the state ($q_{exp}$) from the *open list* with the highest priority (lowest cost) and, unless it fulfils the termination criteria, expands it. The cost, f(q) is the sum of the path cost g(q) and the heuristic cost h(q) as seen in (1).

(1) f(q)=g(q)+h(q)

The goal of the algorithm is to find a path which solves the query in the lowest possible cost (e.g., shortest path), therefore a state with a low cost is assigned high priority. The state that is selected for expansion is then expanded to explore the search space. The expansion to create new offsprings is based on the kinematic constraints of the robot. Each generated offspring needs to pass a validity check to be considered as a feasible candidate. If the new offspring does not pass this check it is discarded as it is either not feasible or it is not able to contribute in the search for the shortest path. If the cell which the state ends up in is free or the cell has a higher cost and the trajectory is collision-free, the new state is added to the *open list* and the cell is updated (this is further described in Section 2.2.4). When all offsprings have been processed the algorithm continues on its next iteration and selects the next state for expansion. This process is repeated iteratively until the goal has been reached or until the *open list* is empty when the algorithm selects a new state for expansion. The termination condition is usually to reach a certain configuration or to be within a region. If the *open list* is empty when the next state is selected for expansion, it means that all of the reachable configurations under the specified resolution have been searched without finding a solution, and therefore no solution exists for the given conditions.
**Algorithm 1** Hybrid A***Input:**$X_{start},X_{goal}$ : Start and goal configurationgrid : GridO : Obstacles    1:**procedure** HA*    2:    $q_{start}=X_{start}$    3:    $grid\left(q_{start}\right).list.insert\left(q_{start}\right)$       ▹ add state to the cell’s list    4:    OpenList=PriorityQueue()    5:    $OpenList.insert(q_{start})$    6:    **while** OpenList!=∅ **do**    7:        $q_{exp}=OpenList.pop\left(\right)$    8:        **if** $q_{exp}\in X_{goal}^{region}$ **then**    9:           **return** $q_{exp}$                      ▹ Solution found    10:        **end if**    11:        $q_{new}=Expand\left(q_{exp},O\right)$    12:        **for each** ${\hat{q}}_{new}\in q_{new}$ **do**    13:           **if** $Valid({\hat{q}}_{new},grid)$ **then**    14:               $grid\left({\hat{q}}_{new}\right).list.insert\left({\hat{q}}_{new}\right)$    15:               $OpenList.insert({\hat{q}}_{new})$    16:           **end if**    17:        **end for**    18:    **end while**    19:    **return** *∅*    20:**end procedure**

### 2.2. Hybrid-A* in the Underwater Domain

The original HA* algorithm was mainly limited to autonomous cars in SE(2). We extend the algorithm to cope with 3D workspaces and therefore planning in SE(3) C-Space. Hence, the algorithm needs to be able to handle the new degrees of freedom: depth (z), roll (φ) and pitch (θ). Due to the nature of sensors used for data gathering and navigational purposes, roll and pitch are treated differently. While submerged, the robot has generally no access to absolute positioning. Instead, the robot often relies on internal sensors to estimate its position. A common approach to estimate the robot’s position is through a doppler velocity log (DVL). A DVL transmits acoustic signals and measures the Doppler shift in the returning signal which can be translated into relative velocity towards the surface the signal was reflected off. However, if the angle of the signal compared to the surface of the medium it reflects off is too high, the signal might be lost as the returning angle of the acoustic signal is not towards the sensor. This causes the sensor to lose *bottom-lock* and not be able to estimate its velocity and hence not position. Therefore, if the angle of pitch is too far from the horizontal plane the position estimation might not work as well as required to follow a planned trajectory. Hence, if a state has a pitch angle larger than the accepted range, the state will be seen as non-valid. While pitching might reduce the quality of such sensors, it is for some vehicles necessary to change depth. Roll, however, is in most cases not desirable and therefore planning in such dimension is not considered.

#### 2.2.1. Expansion of a State Using Motion Primitives

When a state is expanding (line 11 in Algorithm 1), we propose to use a set of motion primitives that defines a discrete set of motions which are feasible for the vehicle. Discretising the robot’s kinematic range into a set of feasible motions reduces the search space and, therefore, lowers the computational time required to solve a query. The set of motion primitives, noted as Φ, consists of *n* different branches (noted ϕ). Each branch represents a continuous motion and consists of *k* intermediate configurations used to check for collision with the known environment. This pre-calculated set of motions only needs to be defined once. All the branches in the set of motion primitives originates from $q_{0}=\left[0,0,0,0,0,0\right]$. Every intermediate configuration ($\left[\phi_{1},..,\phi_{k}\right]$) of a branch can then be transformed to the expanding state ($q_{exp}$) to get its state in the planning frame.

**Figure 2:**
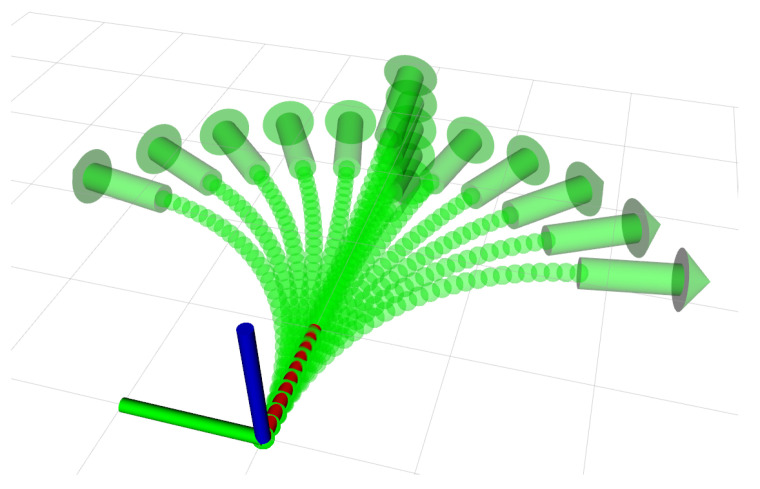
A set of motion primitives in SE(3) is constructed using the kinematic model of a bicycle. This is a discrete representation of the robot’s motion capabilities. The set of motion primitives consists of 1 straight path, 10 curvatures in the horizontal plane and 4 in the vertical.

For this paper, we define the motion primitives such that they can be followed by non-holonomic robots, without hovering capabilities. The motion primitives of the robots are represented using the kinematic model of a bicycle, both for the movement in the horizontal and vertical plane. By using this kinematic model, a set of motion primitives such the one shown in Figure 2 can be created.

#### 2.2.2. Binary Search for a Lower Cost Motion Primitive

**Figure 3:**
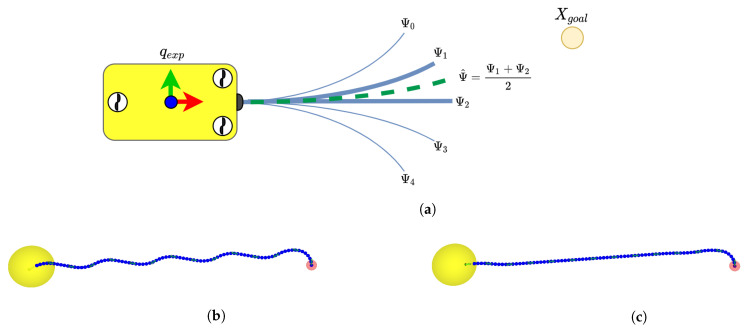
(**a**) When a state is expanding it applies a binary search to find a motion primitive which leads to the state that is closest to the goal within the set of motion primitive’s outer limits. This state should, if within the current capabilities from $q_{exp}$, have a heading towards the goal configuration. (**b**) A path found without using binary search. (**c**) Using binary search can create a path that is heading towards the goal in a straighter path, and therefore also resulting in a shorter path length.

When a state $q_{exp}$ has been expanded to produce new offsprings, we apply a binary search over the branches leading up to the offsprings. This search is used to find a new state, which is the closest (based on the resolution of the search) reachable configuration from $q_{exp}$ to $X_{goal}$ within the motion constraints of the motion primitives. The binary search is recursively applied for *m* iterations and can be seen in Algorithm 2. After *m* iterations, the recursive function returns the branch which results in the state closest to $X_{goal}$. An example of how one iteration of this generates a new branch ($\hat{\Psi }$) can be seen in Figure 3. After the *m* iterations, the search-tree is expanded with the branch found as the most favourable by the binary search. If the motion primitives such as the one pictured in Figure 2 are used, the new branch will reach, if within the outer limits, a heading which is closer towards the goal than the branches in the set of motion primitives. Using the binary search to generate a state closer to the goal will enable the planner to find a path which is reaching the goal region faster as can be seen in Figure 3b,c due to being able to extend a straight path towards the goal.
**Algorithm 2** BinarySearch**Input:**Ψ : Set of branches*m* : iterations    1:**procedure** BinarySearch    2:    $\Psi_{i}=min\_h\_cost\left(\Psi \right)$     ▹ End state of the branch with the lowest heuristic cost    3:    ${\hat{\Psi }}_{1}=\Psi_{i}$    4:    ${\hat{\Psi }}_{2}=min\_h\_cost\left(\Psi_{i-1},\Psi_{i+1}\right)$    5:    ${\hat{\Psi }}_{avg}=average\left({\hat{\Psi }}_{1},{\hat{\Psi }}_{2}\right)$                             ▹ Branch in-between ${\hat{\Psi }}_{1}$ and ${\hat{\Psi }}_{2}$    6:    $\Psi^{\prime }=\left\{{\hat{\Psi }}_{1},{\hat{\Psi }}_{avg},{\hat{\Psi }}_{2}\right\}$    7:    **if** m==0 **then**    8:        **return** $min\_h\_cost(\Psi^{\prime })$    9:    **else**    10:        $BinarySearch(\Psi^{\prime },m-1)$    11:    **end if**    12:**end procedure**

#### 2.2.3. Priority of Expansion

When a new state is created, it is assigned a cost based on the cost to reach the state from the root of the tree and the heuristic cost to reach the goal. If the heuristic is equal or lower than the actual cost of moving from a state to the goal, the path found will be the optimal path [37]. If the heuristic is higher than the cost to reach the goal, the algorithm becomes greedy, giving a larger bias to expand states closer to the goal. Using a weighted heuristic, multiplying h(q) with a weight (ϵ) in (1), can therefore speed up the algorithm but does not guarantee finding the optimal path. Instead, the algorithm can find a solution which is at worst ϵ times longer than the optimal [38]. The higher the weight ϵ, the greedier the search becomes and the less it is focused on exploring the search-space. When ϵ=1, the algorithm becomes a breadth-first search as Dijkstra’s algorithm. The heuristic is calculated using Dubins curves [16]. Dubins curves are used to connect two configurations with the shortest path using a combination of the three segments consisting of constant curvatures Left and/or Right) and/or Straight segments. Dubins curves are originally implemented for 2D. To compensate for the vertical component of the two 3D configurations, we project them into a 2D space. The configurations are separated by the Euclidean distance to incorporate the vertical (z) component before finding the Dubins curves which connects them. Dubins curves as a heuristic are calculated using Open Motion Planning Library (OMPL) [39].

#### 2.2.4. Expanding the Tree with a State

An expanding state will create new offsprings to be potential candidates which can be added to the search tree. The offspring needs to pass a validity check before being added to the tree. During the expansion of a state, each branch that is being expanded must be checked for validity, which includes the state being collision-free and passing the domain-related constraints.

For the previously used application of HA*, the planning has been in a plane and a discrete grid of 3 dimensions (x, y and θ) has been used. As underwater robots operate in a 3D space, the discrete grid needs to include the additional dimensions. However, as previously stated, changing the roll is undesirable for many underwater vehicles and therefore we get a 5D grid consisting of: x, y, z, φ (pitch) and θ (yaw). The grid is a 3D volume consisting of voxels. Each voxel has been divided into two additional dimensions (for pitch and yaw). For consistency throughout the paper, we will refer to *volume* as *grid* and *voxel* as *cell*.

When a new state $q_{exp}$ is created and it passes as being both collision-free and satisfying the domain-related constraints, the algorithm checks if the cell ($q_{cell}$) which $q_{exp}$ has its configuration in is free. If $q_{cell}$ is occupied, an evaluation of the new state is performed to check if it can provide a better solution than the state with the lowest path cost currently occupying the cell. When a state is expanded to the occupied cell, the following three scenarios exist and are handled as the following:

1. $q_{exp}^{g\_cost}=q_{cell}^{g\_cost}$: As $q_{exp}$ could have a slight difference from the ones already occupying the cell which might lead to a better solution the algorithm will allow $q_{exp}$ to be added to the search tree.
2. $q_{exp}^{g\_cost}>q_{cell}^{g\_cost}$: $q_{exp}$: The state is discarded as it is likely to lead to a worse solution.
3. $q_{exp}^{g\_cost}<q_{cell}^{g\_cost}$: The new state finds a shorter path to $q_{cell}$. We can however not change the parent as in A* or Dijkstra’s as this might not comply with the motion constraints of the robot and instead $q_{exp}$ is added to the tree, and the new cost of the cell will now be $q_{exp}^{g\_cost}$ as this is the lowest cost of a state in the cell’s list.

## 3. Improved Replanning by Tree Pruning

When a vehicle is operating autonomously, it does not necessarily have all the information about its surroundings. It is therefore important to be able to map and react to new information about the environment in case such new information requires the vehicle to react in order to, for instance, avoid a collision.

Even if the robot is fully aware of its surroundings before initiating the mission, so that a collision-free and feasible path can be pre-calculated, the robot still needs to verify the validity of such a path during the mission’s execution. Continuous validation of the path helps the vehicle overcome some navigation cumulative errors or even changes in the environment. Hence, no matter the level of knowledge about the environment, it is beneficial for the robot to be able to react to its surroundings. If the robot is following its current calculated path towards the goal, and it is continuously mapping its surroundings using e.g., simultaneous localisation and mapping (SLAM) [40], it might sense an obstacle which invalidates the current path. To avoid collision, the robot needs to react to such an event. When such an event occurs, the robot can handle the situation in two different ways—either the current plan can be discarded and the path planning algorithm can solve the problem from scratch or the current plan can be repaired. The former being the simpler approach, as the planning algorithm can be run as a new instance and solve the start-to-goal query from the current configuration of the robot. The latter, however, can reduce the time or iterations to find a new valid solution. The primary problem when repairing a path which is based on the kinematics of the robot is that connecting two states might not be feasible without complicated manoeuvres. Instead, when planning under kinematic constraints, we apply a branch pruning procedure to enable the re-use of the previously explored paths. Our extension adds a pruning step to HA*. This step prunes the current explored search tree of branches which have been deemed infeasible due to collision with newly discovered obstacles.

### 3.1. Online Mapping and Collision Detection

Obstacles in the environment are uniquely perceivable by the robot when they lie in the detection range of the robot’s sensors. Therefore, as a robot moves through an environment, it incrementally discovers points on the boundary of nearby obstacles. To generate a representation of the surrounding environment online, we adopt a mapping strategy that fuses the robot’s observations into a probabilistic voxel representation. Specifically, we employ the facilities provided by OctoMap [41] to fuse the robot’s range-based data into a 3D occupancy grid map at variable resolution. Voxels in the grid map are initially categorised as unknown, and updated to occupied or free when multiple observations support such hypotheses. OctoMaps efficiently encode the observed environment as an octree data structure, thus optimising memory usage while, at the same time, providing fast access.

**Figure 4:**
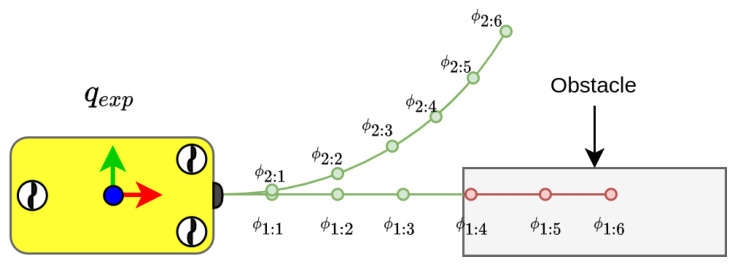
Incremental collision check is performed by traversing the intermediate states of a branch in the motion primitives until either the end state is reached (as in $\psi_{2:6}$) or a collision is found (as in $\psi_{1:4}$).

**Figure 5:**
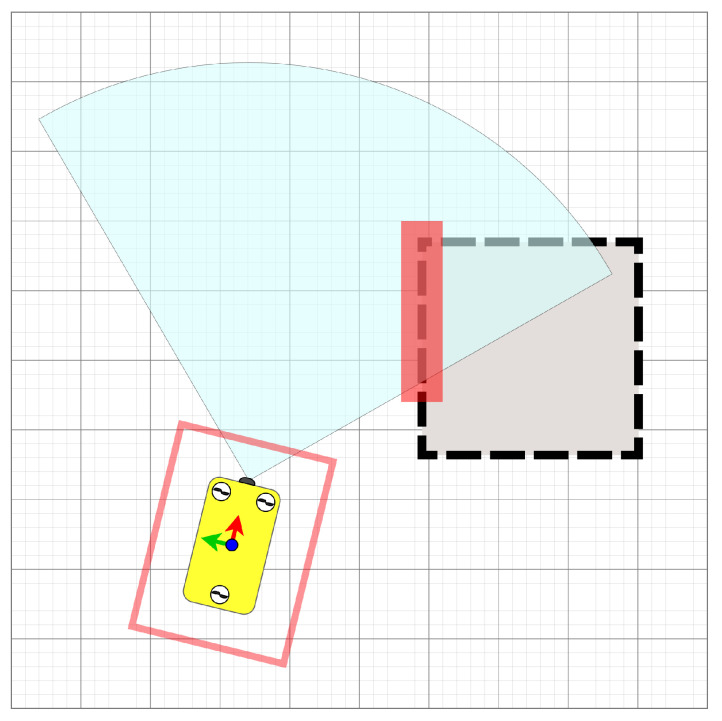
During operation, to compensate for errors in control and sensing, the approach inflates the sensed environment and/or the bounding box of the vehicle. The cells within the red box are added to the map used for collision detection.

The OctoMap representing the sensed environment is periodically updated in the planner. In the map, a bounding box of the robot is used to check for collisions with Flexible Collision Library (FCL) [42]. When a state $q_{exp}$ is expanded, the intermediate state of the branches in the set of motion primitives are incrementally transformed to the frame of $q_{exp}$. If the last intermediate state of a branch is collision-free and fulfils the validity check, the branch is valid and can be added to the tree. In Figure 4, the incremental collision check can be seen. To compensate for errors in vehicle control and noise in the sensing of the map, the environment and/or the bounding box for the robot can be inflated, see Figure 5. Each time the planner’s map is updated, the validity of the current solution is checked; if no longer valid, the planner prunes the tree before solving the query to find a new solution.

### 3.2. Tree Pruning

**Figure 6:**
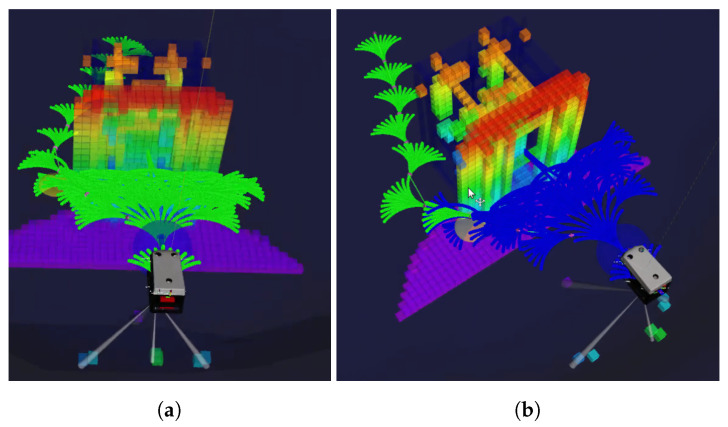
Best viewed in colour. (**a**) The search tree prior to the map being updated is shown in green. With the updated map, the solution is however no longer valid as it intersects with the environment. (**b**) The tree in (**a**) is pruned from branches which are in collision with the environment, the remaining valid tree is shown in blue. The pruned tree is used to initialise the search to find a new solution. The new explored tree required to find a new solution is shown in green.

When operating in unknown environments, with the ability to perform online mapping, the robot can change its path during execution if needed to avoid collision with newly sensed obstacles in the environment. One approach to handle scenarios where the current path is deemed as no longer valid due to collision is to discard the solution and initiate a new planning problem from the current configuration. For geometric planners, approaches on how to repair the path have been proposed, such as ADA* [24] and Field-D* [22], to connect trees which remain valid after removing the states which are in intersection with the environment. However, for planners incorporating the kinematic constraints of the vehicle, connecting a state to another, the right set of controls need to be found, hence making it a more difficult problem to solve. In this paper, we instead apply a tree pruning procedure, which maintains a valid search tree during operation. When the environment is updated, the tree is pruned of the branches the environment intersects with. By keeping a valid search-tree we can reuse previously explored paths and we can solve the query by initiating the HA* with a tree instead of a single root state. This can save time as the algorithm does not have to explore the paths which have already been explored in previous searches. When the currently known map is updated, the solution is checked if it is collision-free. If the path is still valid, the vehicle keeps moving towards the next waypoint. Whenever the robot reaches a waypoint, the state in the tree representing this waypoint is pruned and set as the new root, similar to what Hernández et al. proposed with an RRT* [28] for an AUV operating in SE(2). The prior states are not kept as the tree is based on directed connections between states. A directed connection between states means that if a vehicle can move from $q_{a}$ to $q_{b}$ using a branch from the set of motion primitives, it does not mean that the robot can move from $q_{b}$ to $q_{a}$ using the same connection. If the updated map, however, leads to the solution colliding with the newly sensed obstacles, the algorithm enters the tree pruning step. The algorithm for pruning can be seen in Algorithm 3. The pruning step recursively traverses the tree and discards invalid states. When a connection between a state and its parent state is no longer valid, the corresponding branch will be pruned and as such all states that are dependent on the pruned state are also discarded. When the tree has been pruned, all of the remaining states are valid and the remaining tree is used in the HA* algorithm as an initial search tree. This approach for tree pruning based on new information about the environment has previously been used by Ferguson et al. [32] with Dynamic-RRT. If replanning from scratch (discarding the tree and planning from current configuration) using a deterministic approach, the algorithm will regrow a large portion of the tree which was discarded. Hence, using the pruned tree can remove the need to regrow a largely identical tree, therefore reducing the replanning time. An example of this can be seen in Figure 6 where the path in Figure 6a is no longer collision-free due to new observations of the environment. The tree is then pruned and used to find a new valid solution to the query.
**Algorithm 3** PruneTree**Input:***q* : StateO : Obstacles    1:**procedure** PruneTree    2:    **if** Valid(q,O) **then**    3:        **for** each $\hat{q}\in q_{offsprings}$ **do**    4:           $PruneTree(\hat{q},O)$    5:        **end for**    6:    **else**    7:        DeleteState(q)            ▹ Delete state *q* (and all offsprings recursively) from tree    8:    **end if**    9:**end procedure**

## 4. Tests and Evaluation

In this section, we present comparative results for HA* in both known and unknown environments. All the results were performed in simulations on an i7-7700 CPU @ 2.80 GHz. The approach was implemented using C++ with Robot Operating System (ROS) [43].

### 4.1. Comparison in Known Environments

**Figure 7:**
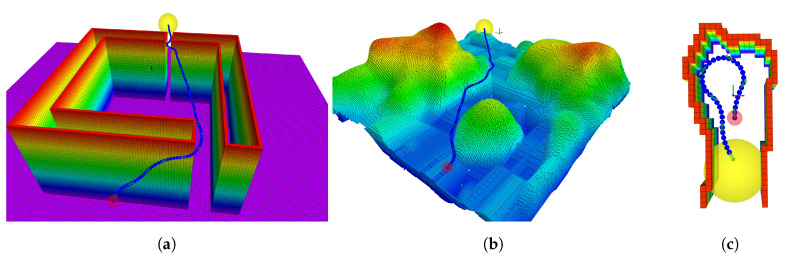
The three known scenarios used to compare our HA* implementation to state-lattice A*, RRT and Dubins-RRT*. The figures include the solution of HA* (The path of intermediate states in blue with green arrows for the configurations of the states). The red sphere shows the start of the query. The yellow sphere is the goal region. (**a**) Scenario 1: Gap. (**b**) Scenario 2: Canyon. (**c**) Scenario 3: Cave (Dead-end).

In this section, we present an evaluation of our HA* algorithm compared to state-lattice A* [12], the RRT [9] and an RRT* that uses Dubins curves [17]. We perform the comparison in the three different environments represented as OcotMaps shown in Figure 7.

The evaluation is performed by running our proposed HA* and state-lattice A* in each test scenario until they find a solution; as both planners are deterministic, they only need to be run once (A deterministic path planning approach will find the same path in the same time when the scenario and parameters are the same). RRT and RRT* are both sampling-based approaches with a stochastic behaviour, thus we run each of them 1000 times and present the mean, median, the minimum and maximum path length of the found solutions. The sampling-based approaches are evaluated with different planning times. They are initially given the same time required by HA* to find a solution, such that we can compare fairly the quality of their solutions (path length) and their success rates. Then, we increase their allowed planning time until they reach a success rate close to 1.0. As the methods are slightly different when operating in 3D, RRT* with Dubins curves is assuming hovering capabilities and diving without change in pitch and state-lattice A* does not scale well into 3D—therefore, this comparison is performed in SE(2). The results of the comparison can be seen in Table 2. The set of motion primitives for HA* consists of 7 uniformly spread branches and the binary search is applied for 3 iterations.

#### 4.1.1. Scenario 1: Gap

The first scenario (see Figure 7a) for the comparison in a known environment is an artificial structure containing two gaps, a smaller and a larger which the robot can go through or it can take the longer path around the structure, the query is ([−30,0,0]→[30,0,0]). State-lattice A* requires longer time and finds a longer solution. Given the same planning time as HA*, neither of the sampling-based approaches manages to find a path which passes through the two gaps but instead circum-navigates the whole obstacle. In #1.6–1.10, the sampling-region for RRT and RRT* is set to a smaller region, forcing the path to go through the two gaps and as seen the success rate severely drops.

#### 4.1.2. Scenario 2: Canyon

In the second test scenario (see Figure 7b), the robot moves through a natural-like terrain, consisting of a canyon and a *rock*. The query ([−25,−10,0]→[25,25,0]) is solved faster by HA* than state-lattice A* and with a lower path length. The sampling-based approaches’ success rate is low given the same time as HA* needs to solve to query. When RRT* with Dubins curves is given roughly 20 times longer planning time than HA*, its success rate gets close to 1.0 but the average length of the solution is roughly 20% longer.

#### 4.1.3. Scenario 3: Cave, Dead-End

The last scenario (see Figure 7c) represents the case where the robot is moving towards a dead-end, thus requiring it to turn back to avoid getting trapped in the cave due to its motion constraints. HA* is able to solve the query $\left(\left[0,-2,\frac{\pi }{2}\right]\to \left[0,-7,-\frac{\pi }{2}\right]\right)$ while state-lattice A* cannot solve it due to its resolution. When the same planning time is given to sampling-based approaches as the time it takes for HA* to solve it, the success rate is low. A drawback in using RRT* with Dubins curves is that the turning radius of the robot is determined by a constant surge speed. This limits the dynamic behaviour of the robot compared to our presented HA* where the set of motion primitives considers a larger range of behaviours. Using a larger set of motions enables HA* to solve queries where a more precise combination of motions are needed to solve the query.

#### 4.1.4. Known Environment—Results

The results from the experimental evaluation in a known environment can be seen in Table 2. Overall, HA* finds shorter paths (higher quality) in less time and with higher success rate than compared approaches. The planning time to solve the queries with a success rate of 1.0 is lower than compared approaches in all cases except for RRT in the gap scenario (see #1.4 in Table 2). However, in #1.4 the quality of the solution, the mean path length, is over twice the length as the path found by HA*. In the scenarios where other approaches manage to find a path length which is shorter than HA* (#2.3, #3.5, #3.6, #3.8 and #3.9) the planning time is higher and the success rate is lower (except in #3.9 where the success rate is also 1.0).

### 4.2. Comparison in Unknown Environments Using Tree Pruning

For the operation in unknown and unexplored environments, we integrate the described methods for mapping, motion planning and tree pruning capabilities with a robot in a UUV Simulator [44]. The simulated robot is equipped with a forward-looking sonar [45] to perceive the environment. We limit the range of the forward-looking sonar to 10 m. The sensor’s observations are fused into a probabilistic map online at 0.5 m resolution. For the simulated scenarios, we apply a weighted heuristic where ϵ=1.5 and use a bounding box for the robot of 2×2×2 metres for collision checking. The set of motion primitives applied is as described in Figure 2, where each branch is 3 m long and has an intermediate state at every 0.25 m. The goal region is a sphere with a radius of 3 m. In this section, we compare our presented approach for replanning using HA* with tree pruning to planning from scratch. When replanning is required, a second instance of HA* is started to solve the problem from the current configuration using the sensed map but not the pruned tree.

**Figure 8:**
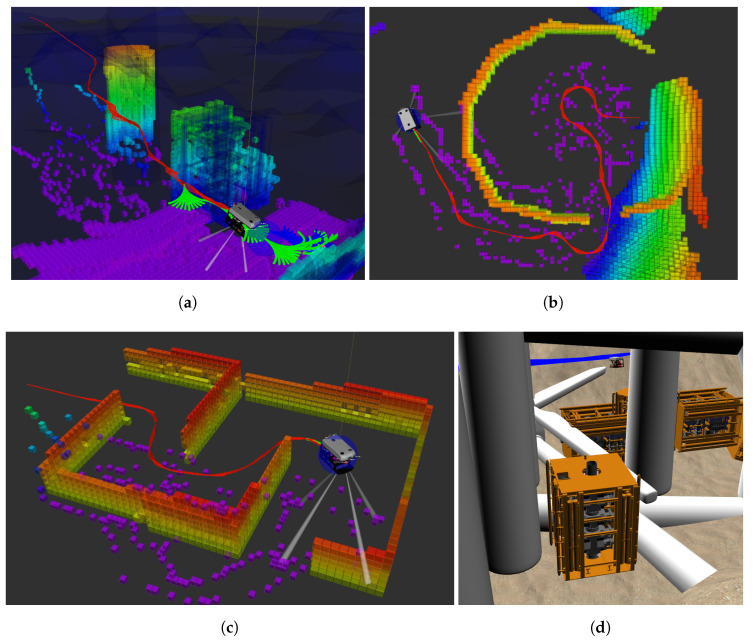
The 4 simulated scenarios. (**a**) Scenario 1: offshore structures. (**b**) Scenario 2: circle/narrow exit. (**c**) Scenario 3: corridor. (**d**) Scenario 4: incident/cluttered. (**a**–**c**) The OctoMap constructed from the data of the forward looking sonar throughout the execution of the plan (red trajectory). (**d**) Shows the Gazebo world using the UUV Simulator, where the robot needs to navigate in a cluttered environment through fallen wind turbines, pillars and other offshore structures. The robot is depicted at the end of the query in all of the images.

The comparison is performed in the following 4 environments (which can be seen in Figure 8):

- Scenario 1: Offshore structures
  The first scenario (see Figure 8a) that is considered consists of two common offshore structures: a blowout preventer (the model for this structure can be seen in Figure 8d) next to the foundation of a wind turbine.
- Scenario 2: Circle/Narrow Exit
  The second scenario (see Figure 8b) is a circular structure with an exit. The robot starts from the inside of the structure and the goal region is on the outside. As such, it will first move straight towards the goal until it finds out that the path is blocked. The set of motion primitives is used without vertical movement in this scenario.
- Scenario 3: Corridor
  The third scenario (see Figure 8c) is navigating through a corridor, where walls partially blocking the inner passage need to be circumnavigated. The set of motion primitives is used without vertical movement in this scenario.
- Scenario 4: Offshore Incident/Cluttered
  The last scenario (see Figure 8d) is a cluttered environment, modelled as an offshore incident with wind turbines which have fallen over next to other offshore structures.

**Figure 9:**
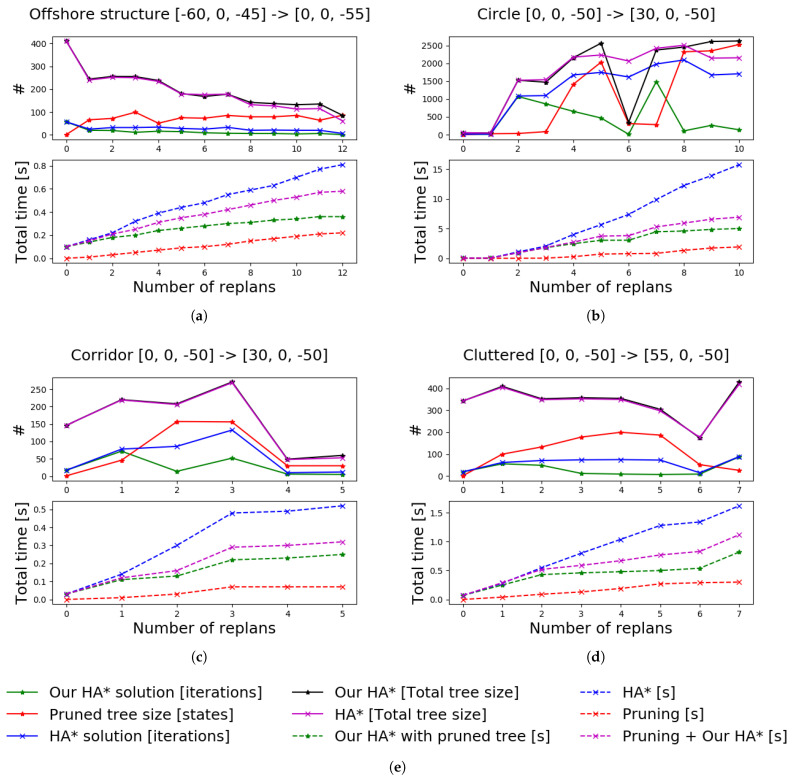
A comparison between our proposed approach for replanning using a pruned tree with HA* compared to HA* replanning from a single root node. Our approach reduces the time spent planning in all considered scenarios. The start position of the robot and the goal are presented, for each scenario, above the corresponding graph. (**a**) Scenario 1: offshore structures. (**b**) Scenario 2: circle/narrow exit. (**c**) Scenario 3: corridor. (**d**) Scenario 4: incident/cluttered. (**e**) Legend to describe the graphs.

In all scenarios, the robot has no initial knowledge about the environment and the two compared approaches use the same parameters and set of motion primitives. The path planner generates the path which the robot follows until the vehicle either reaches the goal or senses an obstacle which intersects with the current plan. If the current plan gets invalidated, the planner prunes the current tree and uses this to plan a new path using our approach. The comparison between planning using the pruned tree and planning from scratch can be seen in Figure 9. The comparison includes the computational time (time for pruning and planning) to solve the query at each occasion replanning is required. In addition, we present the size of the final trees (and the size of the pruned tree) and the number of iterations needed to find a solution. The comparison results in Figure 9 show a significant reduction in computational time by using our proposed approach. In scenario 2 (Figure 9b), it can be seen at the sixth time of replanning that the new path is found after very few iterations using our approach. The pruned tree in such a case contains states which are close to solving the query. Therefore only a few iterations are needed to find a new solution. A similar situation can be seen in Figure 6. These results demonstrate that using a pruned tree instead of replanning from scratch can greatly reduce the computational time spent on planning.

## 5. Conclusions and Future Work

In this paper, we have presented two extensions to hybrid A* for online path planning in 3-dimensional unexplored environments. The first extends HA* to operate in SE(3) with a focus on usage for non-holonomic autonomous underwater vehicles. The second is endowing the planner with tree pruning capabilities to improve operations in unexplored environments. The tree pruning procedure maintains a valid search tree which can be used as an initial search condition when replanning is required. We show that our variant of the HA* planner can find shorter paths, with a higher success rate than RRT, RRT* with Dubins curves and state-lattice A* in known environments. For unexplored environments, we show a consistent reduction in total planning time required to solve a query when using tree pruning compared to replanning from scratch.

As any other search-based planners, the presented variant of the HA* planner is driven by heuristics that indicate the state with the highest priority from where to expand the tree. Such heuristic is counterproductive in environments where local minima exist, i.e., states that rank highly to the heuristic but do not lead to a solution, as it forces search-based planners to explore all local minima states before the planner can find a path around them. One example of such a scenario could be in scenario 1 for known environments, gap (see Figure 7a). If the second gap had been closed, HA* would still have explored the whole space within the structure. Sampling-based approaches would, instead, find a path around it as seen in the results section for such scenario. Improving the algorithm behaviour in such scenarios would greatly benefit the overall applicability of HA*.

For the future, we are planning to integrate the approach with an AUV for real-world tests. The tree pruning procedure in this paper assumes a directed connection between nodes. Extending the tree pruning to include indirect connections could improve the usage for vehicles able to reverse, as it would enable revisiting a previous state. As online planning is critical for autonomous operation for AUVs, we are looking at reducing the total planning time further and alternative approaches to pruning the tree more efficiently.

## Figures and Tables

**Table 1:** Comparison of related work based on the dimension of the C-Space, inclusion of kinematic constraints, suitability for online planning, replanning capabilities in unknown environments and applicability for autonomous underwater vehicles (AUVs). Approaches without replanning capabilities discard their current plan and start the search for a new path without using any prior obtained knowledge about the search-space. The values in bold highlight the desired characteristics for online path planning for a non-holonomic AUV in an unknown 3D environment. ${}^{a}$ Projects the 3D map to 2D to plan in SE(2). ${}^{b}$ Assumes hovering capabilities for the vehicle.

Method	Reference	C-Space	Kinematic Constraints	Online	Replanning	AUV
Search-based						
Field-D*/D*-lite	[22,23]	$R^{2}$	No	**Yes**	**Yes**	No
anytime dynamic A*	[24]	$R^{2}$	No	**Yes**	**Yes**	No
HA*	[13,25,26]	SE(2)	**Yes**	**Yes**	No	No
State-Lattice A*	[12]	SE(2)	**Yes**	**Yes**	No	No
A*	[11]	**SE(3)** ${}^{a}$	**Yes**	No	No	**Yes**
HA*	This paper	**SE(3)**	**Yes**	**Yes**	**Yes**	**Yes**
Sampling-based						
RRT	[27]	SE(2)	**Yes**	**Yes**	No	**Yes**
RRT*	[28]	SE(2)	**Yes**	**Yes**	**Yes**	**Yes**
RRT*	[17]	**SE(3)** ${}^{b}$	**Yes**	**Yes**	**Yes**	**Yes**
SST	[18]	SE(2)	**Yes**	**Yes**	No	**Yes**
RRT*+SST	[19]	SE(2)	**Yes**	**Yes**	No	**Yes**
RRT*+SST	[20]	**SE(3)** ${}^{b}$	**Yes**	**Yes**	No	**Yes**
RRT*+DWA	[21]	*SE*(2)	**Yes**	**Yes**	No	**Yes**
Other						
Non-Lin. Prog.	[29]	SE(2)	**Yes**	**Yes**	No	**Yes**
GA	[7]	$R^{2}$	No	No	No	**Yes**

**Table 2:** Comparison between our presented HA*, state-lattice A*, RRT and RRT* with Dubins curves. The results for the sampling-based approaches are based on 1000 executions. ${}^{b}$ Sampling region forcing the path to go through the gap. ${}^{a}$ Planning time is the average time for RRT to find a solution, allowing planning until one is found in each instance.

			Solution Length [m]				
**#**	**Method**	**Planning Time [s]**	**Mean**	**Median**	**Min**	**Max**	**Success Rate**
**Scenario 1: Gap**							
1.1	HA*	0.147	**66.00**	-	-	-	**1.0**
1.2	State-Lattice A*	0.450589	92.5619	-	-	-	**1.0**
1.3	RRT*	0.147	99.70	99.38	70.48	119.35	0.953
1.4	RRT ${}^{a}$	0.019	135.54	130.43	85.15	248.93	**1.0**
1.5	RRT*	0.02	135.54	131.13	80.34	243.22	**1.0**
1.6	RRT* ${}^{b}$	0.147	77.81	75.36	67.15	102.61	0.089
1.7	RRT ${}^{b}$	0.147	134.71	130.98	69.57	270.03	0.511
1.8	RRT* ${}^{b}$	0.30	77.12	74.84	67.62	101.70	0.177
1.9	RRT* ${}^{b}$	1.00	76.10	73.33	**66.88**	91.90	0.383
1.10	RRT ${}^{b}$	0.12	133.79	129.79	70.20	231.84	0.464
1.11	RRT ${}^{a,b}$	0.26	142.98	139.87	69.26	280.85	**1.0**
**Scenario 2: Canyon**							
2.1	HA*	0.009459	**63.00**	-	-	-	**1.0**
2.2	State-Lattice A*	0.013214	69.84	-	-	-	**1.0**
**#**	**Method**	**Planning Time [s]**	**Mean**	**Median**	**Min**	**Max**	**Success Rate**
2.3	RRT*	0.009459	81.04	73.73	**62.71**	123.32	0.106
2.4	RRT	0.009459	111.75	108.50	64.28	249.34	0.429
2.5	RRT ${}^{a}$	0.01387	122.94	119.05	64.617	245.57	**1.0**
2.6	RRT*	0.20	79.45	72.12	63.44	118.83	0.989
**Scenario 3: Cave (Dead-end)**							
3.1	HA*	0.004985	**18.00**	-	-	-	**1.0**
3.2	State-Lattice A*	No Solution	No Solution	-	-	-	0.0
3.3	RRT*	0.005	19.17	19.17	19.17	19.17	0.001
3.4	RRT*	0.01	18.99	19.05	18.27	19.53	0.01
3.5	RRT*	0.02	18.90	18.76	17.56	21.55	0.067
3.6	RRT*	0.10	19.22	19.17	17.56	22.78	0.978
3.7	RRT	0.005	19.01	19.00	18.64	19.53	0.005
3.8	RRT	0.02	19.29	19.17	17.68	22.61	0.106
3.9	RRT ${}^{a}$	0.035	19.50	19.36	**17.52**	31.45	**1.0**

## Abbreviations

The following abbreviations are used in this manuscript:

ADA*	anytime dynamic A*
AUV	autonomous underwater vehicle
C-Space	configuration space
DVL	doppler velocity log
DWA	dynamic window approach
D*	dynamic A*
EST	expansive space trees
GA	genetic algorithm
HA*	hybrid A*
PRM	probabilistic roadmap
ROS	robot operating system
ROV	remotely operated vehicle
RRT	rapid-exploring random tree
RRT*	asymptotic optimal RRT
FCL	Flexible Collision Library
SLAM	simultaneous localisation and mapping
SST	stable sparse-RRT
